# A high-resolution melting approach for the simultaneous differentiation of five human babesiosis–causing* Babesia* species

**DOI:** 10.1186/s13071-023-05839-5

**Published:** 2023-08-28

**Authors:** Yanbo Wang, Shangdi Zhang, Xiaoyun Li, Yueli Nian, Xinyue Liu, Junlong Liu, Hong Yin, Guiquan Guan, Jinming Wang

**Affiliations:** 1 State Key Laboratory for Animal Disease Control and Prevention, Key Laboratory of Veterinary Parasitology of Gansu Province, Lanzhou Veterinary Research Institute, Chinese Academy of Agricultural Science, Lanzhou, Gansu People’s Republic of China; 2 The Second Hospital of Lanzhou University, Lanzhou, People’s Republic of China; 3 Jiangsu Co-Innovation Center for the Prevention and Control of Important Animal Infectious Disease and Zoonoses, Yangzhou University, Yangzhou, 225009 China

**Keywords:** Human babesiosis, *Babesia*, *Babesia crassa–*like, High-resolution melting, Transfusion-transmitted babesiosis

## Abstract

**Background:**

Six species of apicomplexan parasites of the genus* Babesia*, namely *B. microti*, *B. divergens*, *B. duncani*, *B. motasi*, *B. crassa–*like and *B. venatorum*, are considered to be the primary causal agents of human babesiosis in endemic areas. These six species possess variable degrees of virulence for their primary hosts. Therefore, the accurate identification of these species is critical for the adoption of appropriate therapeutic strategies.

**Methods:**

We developed a real-time PCR–high-resolution melting (qPCR-HRM) approach targeting 18S ribosomal RNA gene of five *Babesia* spp. based on melting temperature (*T*_m_) and genotype confidence percentage values. This approach was then evaluated using 429 blood samples collected from patients with a history of tick bites, 120 DNA samples mixed with plasmids and 80 laboratory-infected animal samples.

**Results:**

The sensitivity and specificity of the proposed qPCR-HRM method were 95% and 100%, respectively, and the detection limit was 1–100 copies of the plasmid with the cloned target gene. The detection level depended on the species of *Babesia* analyzed. The primers designed in this study ensured not only the high interspecific specificity of our proposed method but also a high versatility for different isolates from the same species worldwide. Additionally, the *T*m obtained from the prepared plasmid standard is theoretically suitable for identifying isolates of all known sequences of the five *Babesia* species.

**Conclusions:**

The developed detection method provides a useful tool for the epidemiological investigation of human babesiosis and pre-transfusion screening.

**Graphical Abstract:**

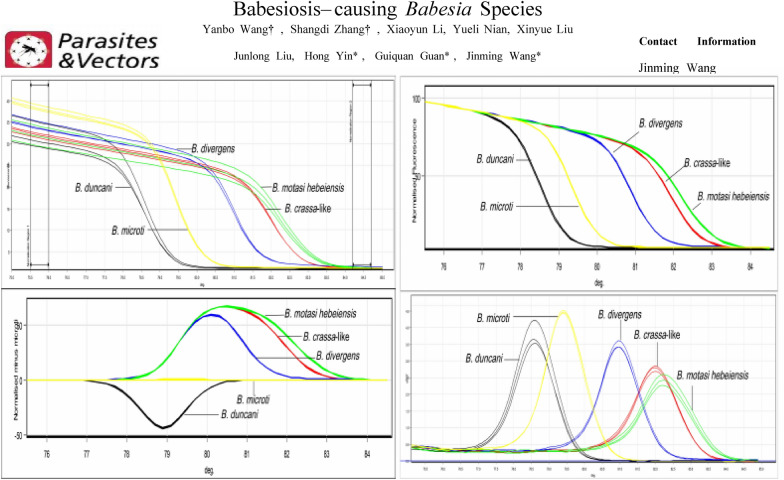

**Supplementary Information:**

The online version contains supplementary material available at 10.1186/s13071-023-05839-5.

## Background

Human babesiosis caused by *Babesia* species, which are blood protozoa belonging to the family Babesiidae (Order Piroplasmida; Phylum Apicomplexa) [1], is a re-emerging tick-borne disease. Cases of transfusion-transmitted and naturally acquired *Babesia* infection in humans represent a serious public health problem and have been frequently reported worldwide in recent years [2, 3]. The pathogens responsible for human babesiosis are predominantly transmitted via tick bites and blood transfusion and rarely via perinatal routes and organ transplantation [4–8]. To date, more than 100 *Babesia* species have been identified worldwide [9].

*Babesia* spp. were previously believed to only infect wild and domestic animals. However, in 1957, the first case of human infection with *Babesia* was reported in Yugoslavia [10], and the disease has gradually become a new global parasitic disease in humans, with the potential to be a health threat to people with weaker immune systems. Six *Babesia* species, namely *B. microti*, *B. venatorum*, *B. divergens*, *B. duncani*, *B. motasi*, and *B. crassa–*like, are well known as the major pathogenic agents responsible for human babesiosis [11, 12]. The majority of *Babesia* cases in the USA are caused by *B. microti*, in areas where the disease is endemic, but *B.* *duncani* and *B. divergens* are also distributed in the USA [13]. In Europe, *B. divergens* is mainly found in France and Ireland [12, 14], *B. venatorum* has been reported in Austria, Germany, Italy and Sweden [14–16] and *B. crassa–*like infections have been reported in Slovenia and France [17, 18]. In Asia, *B. venatorum* and *B. crassa–*like are mainly reported in China [19, 20]. However, *B. microti* cases have been reported in southwestern China along the Myanmar border, Taiwan and Japan [21–25]. *Babesia motasi* strain KO1 (DQ3466955) is distributed in Korea [12, 26]. The sequences of *B. motasi* KO1 and *Babesia* sp. KCDC-1 (MK930513) strains isolated from two human cases in South Korea showed great similarity with the sequence of the *B. motasi* *hebeiensis* strain in China [26]. It has also been observed that *B*. *duncani*, which was first reported in Washington State, USA, is now prevalent in Canada [27–29].

People with a functioning immune system may remain asymptomatic post-*Babesia* infection, although intermittent and low-grade parasitemia can occasionally last for > 2 years [30, 31]. Thus, *Babesia*-infected patients who are asymptomatic may spread the pathogen to others via blood transfusion or organ transplantation [32]. In the USA, parasitic infections related to blood transfusions are almost entirely caused by *Babesia* [33]. It has also been observed that transfusion-transmitted babesiosis causes severe complications and death in approximately one of every five cases [30]. However, in human babesiosis-epidemic areas, blood screening for babesiosis is still lacking or only performed at a limited number of blood collection centers [34].

The diagnostic techniques currently used for *Babesia* (natural hosts and animal models) detection include microscopic examination (ME), immunoassays and molecular techniques [2]. Although the traditional ME has a high specificity, it is characterized by a low sensitivity and requires highly experienced microscopists to carry out the analysis. Common molecular techniques, such as nucleic acid detection, perform well but are relatively time-consuming, and some existing nucleic acid testing techniques for diagnosing babesiosis have high requirements for instruments [35–37]. The immunological detection method is efficient, time-saving and sensitive; however, false positive results caused by cross-reaction often occur during the detection process, and only one pathogen can be detected at a time. The simultaneous detection of several species is a labor-intensive process, and distinguishing current infections from past infections using serological tests is still challenging [38, 39]. While real-time PCR (qPCR)-based techniques, with high specificity and sensitivity, have been proposed for the detection of babesiosis, the number of *Babesia* species that can be differentially diagnosed at the same time using this technique is limited [40–43]. Current diagnostic strategies are typically used to detect major species, notably *B. microti* and *B. divergens*. In contrast, methods with high sensitivity and specificity are lacking for low-prevalence species, such as *B. crassa–*like and *B. motasi* [18]. The true incidence and distribution of human babesiosis may be much higher than the currently reported levels [17, 44].

In areas where multiple *Babesia* spp. are co-endemic, species differentiation is critical for guiding appropriate treatment and monitoring. Tick vectors commonly transmit different *Babesia* species, leading to the simultaneous occurrence of several types of *Babesia* infections in some areas, which not only poses great difficulties in preventing and controlling the resulting diseases and establishing accurate detection methods, but leads to incorrect classification and low detection rates. Notably, the pathogenicity and reproductive ability of *B. duncani* are superior to those of *B. microti* [45], and the former is also more resistant to currently recommended anti-*Babesia* drugs [46]. Therefore, in the future, different treatment methods should be selected according to the virulence and drug resistance of the *Babesia* species.

In this study, we designed a single-round qPCR-high-resolution melting (qPCR–HRM) assay using a single pair of primers and investigated the performance of this assay in simultaneously identifying five zoonotic *Babesia* species, namely *B. microti*, *B. divergens*, *B. duncani*, *B. motasi hebeiensis* and *B. crassa–*like. The 18S ribosomal RNA (rRNA) gene, which contains the highly conserved and variable regions of *Babesia*, was selected as the target amplification region of the qPCR-HRM. The designed primers of HRM do not allow cross-reaction with other zoonotic pathogens, such as *Toxoplasma gondii*. Specifically, the HRM approach is based on the principle that during the dissociation of double-stranded DNA into single-stranded DNA, as the temperature increases, the melting curve and melting temperature (*T*_m_) can be generated by monitoring the fluorescence of a dye in the double-stranded DNA. Different melting curves, different *T*_m_ values, and different genotype confidence percentage (GCP) values can thus be obtained based on differences in the amplicon compositions of the different species [47]. It has been reported that a higher GCP value is indicative of a higher degree of nucleic acid sequence similarity between the sample and positive control. Based on our results, this assay can be used for the differential diagnosis and epidemiological investigation of babesiosis in endemic areas.

## Methods

### Preparation of the plasmid standard

Plasmids, each bearing 18S rRNA gene sequences of *B. microti* (KF410825), *B. divergens* (FJ944826), *B. duncani* (HQ285838), *B. motasi* *hebeiensis* (DQ159074.1) or *B. crassa–*like (AY260176), respectively, were used as positive controls to assess the sensitivity and specificity of the newly developed qPCR-HRM detection method. The plasmids with a cloned target gene of *B. microti* Hebei or *B. divergens* were synthesized by Tsingke Biotech Co. (Beijing, China). The strains of *B. motasi hebeiensis* and *B. duncani* were obtained from the Vectors and Vector-Borne Diseases (VVBD) Laboratory, Lanzhou Veterinary Research Institute (LVRI), China. Briefly, the PCR-amplified fragments of each *Babesia* strain were purified using the Zymolean™ Gel DNA Recovery Kit (ZYMO Research Corp., Los Angeles, CA, USA), cloned into the pGEM-T Easy vector (Promega, Madison, WI, USA) and transformed into *Escherichia coli* DH5α competent cells (Takara Biotech Co., Ltd., Dalian, China). Three clones from each sample were then selected and cultured for 12 h in Luria–Bertani (3 ml) medium containing 100 μg/ml ampicillin (Merck, Darmstadt, Germany). Plasmid extraction was then performed using a Plasmid Miniprep Kit (Axygen Scientific Inc., Union City, CA, USA). This was followed by sequencing using the Big Dye Terminator Mix (Genscript, Nanjing, China). Plasmid concentration was measured using a NanoDrop 2000 spectrophotometer (NanoDrop Technologies, Wilmington, DE, USA). The prepared plasmids were then diluted to 10^7^–10^−1^ copies/µl using enzyme-free water (Axygen Scientific Inc.).

### Preparation of laboratory-infected animal specimens and DNA samples

Six-month-old sheep, negative for piroplasm infection as determined via microscopy and PCR, were purchased from Jingtai County, Gansu Province, China [48]. Splenectomy was performed on the sheep 1 month before the experiment. The strains of *B. motasi* *hebeiensis* and *B. duncani* were obtained from the VVBD Laboratory. In brief, to prepare the infected animals, a suspension of *B. motasi* *hebeiensis*, preserved in liquid nitrogen, was rapidly thawed in water at 37 °C, and a 50-ml (approx. 1 × 10^9^ infected erythrocytes) aliquot of this solution was injected into the sheep via the jugular vein.

Lakeview Golden (LVG) Syrian hamsters (age: 3 months) were purchased from the Beijing Vital River Laboratory Animal Technology Co., Ltd (Beijing, China). Similar to the infected sheep, after the rapid resuscitation of frozen *B. duncani* in a water bath at 37 °C, five hamsters were inoculated intraperitoneally with 200 μl of the *B. duncani* suspension (approx. 2.0 × 10^6^ infected red blood cells [iRBCs]).

At 4 and 7 days after the inoculation of the hamsters and sheep, respectively, blood samples were collected for the preparation of smears. After Giemsa staining of the blood smears, parasitemia was calculated by ME. Approximately 3000 RBCs were counted per blood smear to calculate the percentage of parasitized RBCs. When parasitemia varied in the range of 8% to 10%, whole blood samples, with different parasitemia levels, were collected in tubes containing ethylenediaminetetraacetic acid (EDTA) anticoagulant. Total DNA was extracted from 200 μl of these blood samples using a commercial DNA extraction kit (QIAamp DNA Blood Mini Kit; Qiagen, Hilden, Germany) according to the manufacturer’s instructions.

The DNA of *T. gondii*, and *Trypanosoma evansi* were obtained from the blood of infected animals collected in the VVBD laboratory. In the specificity experiments, the DNA was directly amplified as a template without any treatment. The negative control DNA was isolated from whole blood obtained from healthy humans, sheep and piroplasm-free tick tissue and examined by ME of blood smears and by nested PCR [48]. A NanoDrop 2000 spectrophotometer (NanoDrop Technologies) was used to assess DNA concentration. The extracted DNA was then stored at − 20 °C until use.

These experiments were approved by the LVRI Animal Ethics Committee of the Chinese Academy of Agricultural Sciences. All procedures were performed in accordance with the Animal Ethics Procedures and Guidelines of the People's Republic of China.

### Preparation of DNA samples mixed with plasmids

The plasmids with a cloned target gene of *B. microti* or *B. divergens* were added to DNA samples from healthy individuals or ticks at different concentrations to prepare experimentally infected (positive) control samples. The number of plasmid copies added to obtain the positive samples ranged from 1 to 10^4^ copy number(s)/μl.

### Collection of clinical samples

Blood samples were collected from 492 patients with a history of tick bite who had visited the Second Hospital of Lanzhou University between May 2017 and July 2019 and resided in the Gannan Tibetan Autonomous Prefecture (Gansu, China). All clinical samples were collected using approved protocols and after obtaining written informed consent from each patient. Total DNA was extracted from 200 μl of each clinical blood sample using the QIAamp DNA Blood Mini Kit (Qiagen).

### Target gene sequence selection and primer design

For this study, we designed primers with high specificity for distinguishing and identifying *Babesia* species. The 18S rRNA gene sequences of five *Babesia* species identified in different countries based on the NCBI database were analyzed using the DNAMAN 9.0 software package (Lynnon Biosoft, Vaudreuil-Dorion, QC, Canada), and the homology of the different isolates of each *Babesia* species was analyzed using MEGA version 7 software. The corresponding fragments of the same *Babesia* species from different regions that showed 90–100% sequence homology were screened out. There were several differences in base length between these common fragments of the five *Babesia* species (Additional file 1: Fig. S1; Additional file 2: Fig. S2). To distinguish the five *Babesia* spp., we designed universal primers using Primer v.5.0 software (PREMIER Biosoft, San Francisco, CA, USA) targeting common fragments for the qPCR-HRM analysis.

### Evaluation of primer efficiency at various annealing temperatures

To ensure that the primers could simultaneously amplify the target regions of the 18S rRNA gene of all five *Babesia* spp. without producing nonspecific bands or primer dimers, both of which would interfere with the interpretation of the results in subsequent qPCR-HRM analysis, we performed a series of gradient PCR assays to determine the optimal annealing temperature for the primers. Briefly, the PCR mix (20 μl) containing the Premix Taq DNA polymerase (10 μl; Takara), DNA template (1 μl), each primer (10 μM/μl) and RNase-free water (7 μl) was prepared, and gradient PCR was then performed using a T100™ Thermal Cycler (Bio-Rad Laboratories, Hercules, CA, USA). Thereafter, the mixture was heated at 95 °C for 2 min, followed by 40 cycles of denaturation at 95 °C for 30 s, annealing at a gradient temperature of 54 °C to 61 °C for 30 s, extension at 72 °C for 30 s, with a final extension at 72 °C for 5 min. After gradient PCR amplification, the products were resolved by electrophoresis in 1.5% (w/v) agarose gels to evaluate primer specificity and amplification efficiency at the different annealing temperatures.

### qPCR-HRM analysis

The qPCR amplification was performed in a 20-µl reaction solution containing the forward and reverse primers(10 nM), Forget-me-Not™ qPCR Master Mix (10 μl; Biotium, Hayward, CA, USA), 40× template buffer (0.5 μl), RNase-free water (6.5 μl) and the DNA sample (1 µl). The qPCR-HRM assays were performed using a Rotor-Gene Q6000 Real-Time PCR system (Qiagen) with an initial denaturation step at 95 °C for 2 min, followed by 45 cycles of 95 °C for 5 s and 55 °C for 30 s. HRM was performed from 75 °C to 85 °C using a 0.2 °C step with a 2-s hold time at each acquisition step. Finally, the test results were analyzed using the High-Resolution Melt software v.2.3.1 program (Qiagen), which automatically generates the melting curve, *T*_m_ and GCP values.

### Evaluation of the analytical specificity and sensitivity of the qPCR–HRM approach

The specificity of the assay was assessed using 20 ng/μl plasmids with a cloned target gene of zoonotic *Babesia*, including from *B. divergens*, *B. motasi* *hebeiensis*, *B. crassa–*like, *B. duncani* and *B. microti*, with the samples diluted by DNA extracted from blood samples collected from healthy humans, to ensure that a pair of primers could be used to accurately distinguish the five *Babesia* spp. in a single reaction. Five replicates were prepared for each plasmid, and the standard *T*_m_ and GCP values of each *Babesia* strain were obtained based on five independent qPCR-HRM experiments. The specificity of this assay was also evaluated using the DNA extracted from other organisms, such as *T. gondii*, *T. evansi*, ticks and hamsters. Genomic DNA extracted from whole blood obtained from healthy humans was used as a negative control.

Analytical sensitivity was assessed using tenfold serial dilutions of the five plasmid DNA standards, ranging from 1 to 10^7^ copy number(s)/μl. This process was repeated 3 times to ensure the accuracy of the results. Standard curves were then plotted using the High-Resolution Melt software v.2.3.1 (Qiagen) to evaluate the efficiency of the assay.

### Evaluation of qPCR-HRM assay using experimental and clinical samples

The performance of the qPCR-HRM assay was evaluated using standard positive samples (experimentally infected animal specimens, DNA samples mixed with plasmids and plasmids), as well as clinical samples (Table 1). The presence of *B. duncani* and *B. motasi* *hebeiensis* in the collected blood samples was detected by microscopy. The previously established nested qPCR method for specific detection of *B. duncani* was used to detect 80 DNA samples mixed with plasmids and 80 laboratory-infected animal samples, and to compare detection performance with the qPCR-HRM assay for detecting *B. duncani*. A total of 492 clinical samples, 120 DNA samples mixed with plasmids, and 80 laboratory-infected animal samples were also randomized, relabeled and blindly detected using the qPCR-HRM method. Simultaneously, the qPCR products of all the samples were purified using a gel recovery kit, and 18S rRNA gene sequencing was performed to verify the qPCR-HRM results.

**Table 1:** Classification of test samples

Type of samples	*Babesia* species	Number of specimens
Spiked DNA samples by the plasmids	*B. microti*, *B. divergens*, *B. crassa–*like	120
Animal DNA samples	*B. duncani*, *B. motasi hebeinesis*	80
Clinical samples	–	492

For mixed plasmid DNA samples and animal DNA samples, there were 40 specimens for each *Babesia* species

### Statistical analysis

One-way analysis of variance (ANOVA) was performed using SPSS software (IBM, Armonk, NY, USA) to analyze whether there were significant differences in the *T*_m_ values corresponding to the five *Babesia* species. The *T*_m_ values of *B. crassa–*like and *B. motasi* *hebeiensis* were further analyzed by performing a t-test.

## Results

### Primer design and testing

**Fig. 1:**
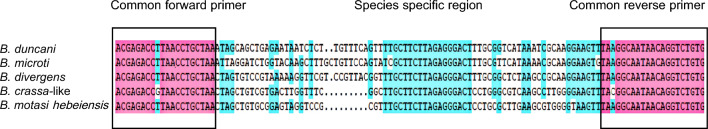
Sequence alignment of common sequences of five *Babesia* spp. amplified by the universal primers used in this study. The sequence for the conserved region of the 18S rRNA common to the *Babesia* spp. is indicated in red. The sequence for the 18S rRNA variant region common to the *Babesia* spp. is indicated in blue. The white portion is the differential base of the 18S rRNA of the five *Babesia* species. Dots represent the base missing in this region of the strain compared to other species. rRNA, Ribosomal RNA

A pair of universal primers (*Babesia*-3F: 5′-ACG AGA CCT TAA CCT GCT AA-3′, *Babesia*-3R: 5′-CAC AGA CCT GTT ATT GCC TTA-3′) was selected to amplify the target sequence located in the 18S rRNA gene of the *Babesia* spp. by screening multiple primer pairs (Fig. 1). According to the Basic Local Alignment Search Tool (BLAST) analysis of the primers, the PCR products varied in length from 118 to 127 bp. The upstream and downstream primers target the conserved regions on both sides of the V3 hypervariable region of the 18S rRNA gene of *Babesia* spp., allowing species differentiation via melting curve analysis. Electrophoresis results obtained at an annealing temperature of 55 °C showed the appearance of a strong band, without a nonspecific band in the negative control (Additional file 3: Fig. S3).

### Verification of specificity

**Fig. 2:**
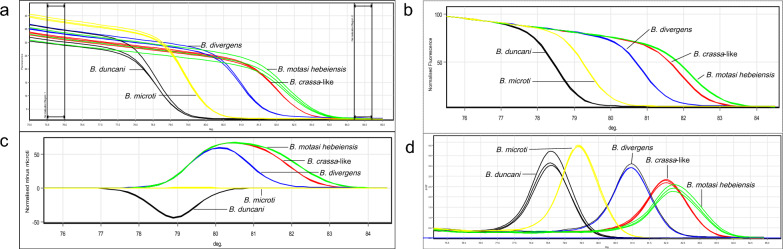
Detection and discrimination of the five *Babesia* spp. **a** Raw data from melt curve analysis, **b** normalized HRM plots for 18S rRNA amplicon, **c** normalized difference curves, **d** derivative plot analyses. The melting curve and *T*_m_ for each species can be very well discerned. HRM, High-resolution melting; *T*_m_, melting temperature

The qPCR-HRM assay successfully distinguished all five *Babesia* spp. considered in this study. Specifically, as shown in Fig. 2, the melting curve of different *Babesia* species could be clearly separated, and the *T*_m_ of each species could be determined. No melting curves were observed for DNA samples belonging to non-*Babesia* pathogens. Further, the negative control containing the host, background DNA did not exhibit amplification. Thus, the *T*_m_ and GCP values obtained could be used as reference standards for the identification of *Babesia* spp. (Table 2). The *Babesia* species were also identified based on similarities in standard curves and *T*_m_ values between the analyzed samples and reference samples.

**Table 2:** Standard melting temperature and genotype confidence percentage values of five *Babesia* species in the real-time PCR–high-resolution melting analysis

*Babesia* spp.	*T*_m_ value range (°C)	GCP
*B. duncani*	78.55 ± 0.08	99.72 ± 0.21
*B. microti*	79.30 ± 0.05	99.79 ± 0.19
*B. divergens*	80.90 ± 0.07	99.61 ± 0.22
*B. crassa–*like	81.99 ± 0.08	99.16 ± 0.48
*B. motasi* *hebeiensis*	82.28 ± 0.05	99.35 ± 0.42

Values are presented as the mean ± standard deviation (SD)

*GCP* Genotype confidence percentage, *T*_*m*_ melting temperature

### Verification of sensitivity

**Fig. 3:**
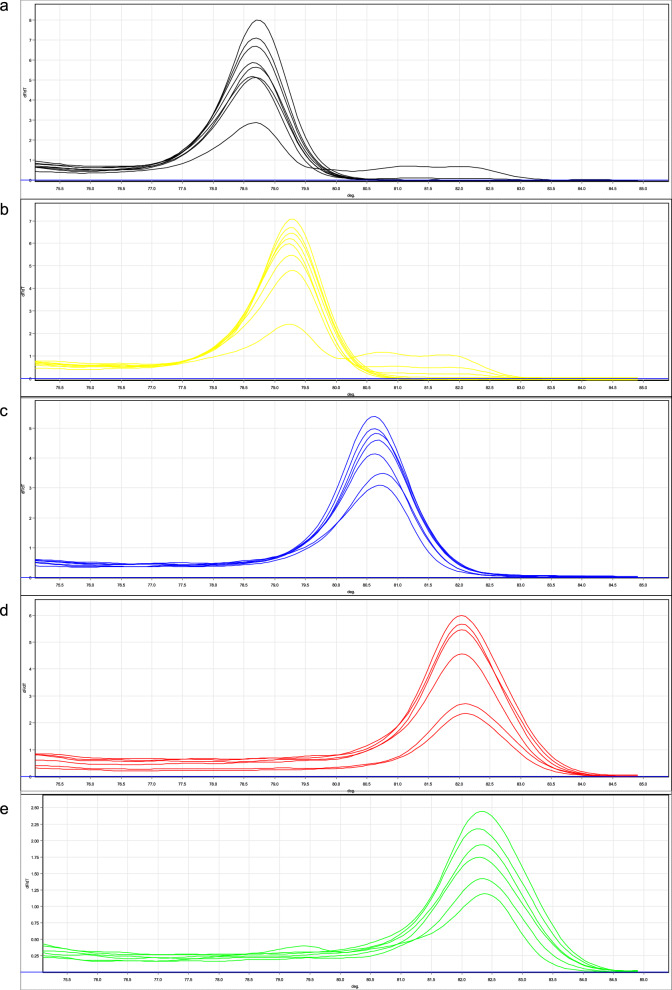
Derivative melting curve of plasmids of *Babesia* with tenfold serial dilution. **a** *B. duncani*, **b** *B. microti*, **c** *B. divergens*, **d** *B. crassa-*like, **e** *B. motasi* *hebeiensis*

The detection limit of the qPCR-HRM assay was evaluated using tenfold serially diluted plasmid DNA samples from the five *Babesia* spp. in three independent reactions, with the concentrations ranging from 1 to 10^7^ copies/µl. The detection limit of the assay for *B. microti* was one copy, whereas for *B. divergens* and *B. duncani,* it was 10 copies. Both *B. motasi* *hebeiensis* and *B. crassa–*like could be detected at as low as 100 copies of the target sequence (Fig. 3).

### Test on the clinical samples

The discriminatory power of this assay was assessed using a blinded test comprising 200 *Babesia*-positive samples (DNA samples mixed with plasmids and laboratory-infected animal samples) and 492 clinical samples. The observed sensitivity and specificity of the qPCR-HRM assay were 95% and 100%, respectively. Four *B. divergens*-specimens were detected in clinical samples from patients with a history of tick bites (Table 3). The results also showed that the qPCR-HRM approach can be used to detect *Babesia* in different host samples, such as ticks, humans and rodents, and no cross-reaction with host DNA was observed. In addition, samples containing different plasmid copy numbers of *Babesia* were successfully detected. Taken together, these observations indicated that the qPCR-HRM assay can specifically amplify the 18S rRNA gene of *Babesia* spp. The melting curves of the DNA samples extracted from different batches showed high repeatability, and even though there was a slight change in the *T*_*m*_ values, no change in the melting peak shape or in the *T*_*m*_ gap between the samples was observed. The results of one-way ANOVA showed significant differences in the *T*_*m*_ among the five *Babesia* spp. (*p* < 0.0001), sufficiently demonstrating that the qPCR-HRM approach can be used to clearly distinguish *Babesia* species. In the melting curve analysis, the GCP value of the automatic analysis results was 90–100%, further indicating that the species differentiation results are quite convincing.

**Table 3:** Analysis results of clinical samples and laboratory-infected samples

*Babesia* species	Results	*T*_*m*_ value range (°C)	GCP
*B. duncani*	38/40	78.59 ± 0.12	98.09 ± 1.18
*B. microti*	40/40	79.30 ± 0.04	98.81 ± 0.49
*B. divergens*	39/40	80.90 ± 0.11	97.91 ± 1.62
*B. crassa-*like	37/40	82.02 ± 0.10	97.32 ± 1.73
*B. motasi* *hebeiensis*	36/40	82.30 ± 0.06	98.40 ± 1.19
Clinical sample	4/492	80.92 ± 0.09	92.45 ± 1.38

Values are presented as the mean ± SD

**Fig. 4:**
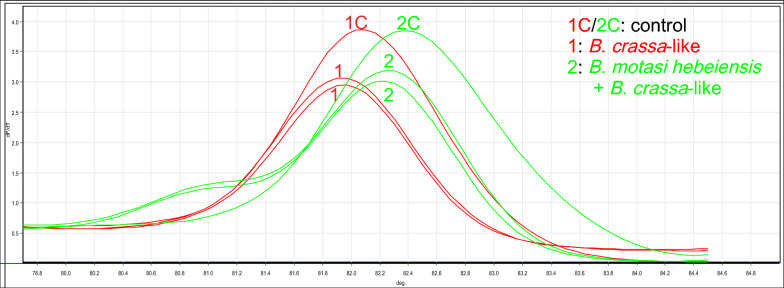
Differential diagnosis of *B. crassa-*like and *B. motasi* *hebeiensis*.* 1C* PCR product of plasmid of *B. crassa-*like,* 2C* PCR product of plasmid of *B. motasi* Hebei,* 1* mixed samples of PCR products of plasmid and DNA of *B. crassa-*like,* 2* mixed sample of PCR product of *B. motasi* *hebeiensis* DNA and PCR product of *B. crassa-*like plasmid. The qPCR product of the plasmid of *B. crassa-*like was mixed with the qPCR products of two unknown *Babesia* samples at a volume of 1:1, and then HRM analysis was performed

Even though the *T*_*m*_ produced by the target sequences of *B. crassa–*like and *B. motasi* *hebeiensis* were relatively similar, these species could still be clearly distinguished given that the* p*-value obtained from the t-test between their *T*_*m*_ values was < 0.0001. Additionally, *B. crassa–*like and *B. motasi* *hebeiensis* could be distinguished based on the shape of the melting curves of the mixed samples. The qPCR product of the plasmid standard of *B. crassa–*like was mixed with the qPCR products of DNA samples of *B. crassa–*like or *B. motasi* *hebeiensis* at a 1:1 volume ratio. The HRM analysis was repeated, and it was observed that the melting curve of *B. motasi* *hebeiensis* formed a double peak, whereas that of *B. crassa–*like formed a unimodal peak (Fig. 4).

The ability of our newly developed method to detect *B. duncani* was evaluated through comparison with the results from microscopy and nested qPCR using 120 DNA samples mixed with plasmids and 80 laboratory-infected animal samples. The results obtained indicated that our proposed method, qPCR-HRM, shows good performance in *B. duncani* detection (Table 4).

**Table 4:** Comparison of the performance of microscopy, nested real-time PCR and the newly developed real-time PCR–high-resolution melting method for the detection of *B. duncani*

Results	Detection method		
	Microscopy	qPCR-HRM	Nested-qPCR
True positive	35/40	39/40	40/40
False positive	2	0	0
True negative	38/40	160/160	160/160
False negative	5	1	0
Specificity (%)	95	100.0	100.0
Sensitivity (%)	87.5	97.5	100.0

*qPCR-HRM* Real-time PCR–high-resolution melting

## Discussion

In recent decades, there has been a gradual increase in the number of human babesiosis cases, largely due to increased human activities in *Babesia* endemic areas, insufficient public awareness regarding human babesiosis, expansion in the range of major tick vectors and climate change. Existing technologies for *Babesia* spp. detection suffer from several limitations [44]. At the present time, the gold standard for the confirmation of babesiosis infection is ME [49], but several *Babesia* spp. that infect humans are not only morphologically similar to *Plasmodium* but also similar to each other [2]. The low parasitemia level obtained may result in these samples being misdiagnosed as false negatives during ME [50]. Several PCR-based assays for the detection of *Babesia* are available, such as droplet digital PCR, cobas® *Babesia* (Roche Molecular Systems Inc., Branchburg, NJ, USA) and a licensed nucleic acid test targeting the *Babesia* 18S rRNA gene [35–37]. Although these methods have high sensitivity and specificity and can be used to detect multiple pathogens simultaneously, a major disadvantage that limits their applicability in babesiosis-endemic countries is that they require complex and expensive instruments. Fluorescence in situ hybridization can also be used to detect active infections; however, this method has shown limited performance in detecting hypoparasitemia during the early stages of infection [51]. Immunofluorescence assays, enzyme-linked immunosorbent assays and multiplex bead format assays can also be used to detect antibodies produced within 1 week of infection. However, the antibodies produced by several *Babesia* spp. in the host appear to be different and, therefore, multiple analyses are required to rule out *Babesia* infection, which is a laborious process [52, 53]. *Babesia* antibodies are usually detectable in the blood for several months after infection remission, and distinguishing between current and previous infections is still challenging [50, 54, 55]. Additionally, these above-mentioned diagnostic methods do not include the specific detection of *B. crassa–*like and *B. motasi* *hebeiensis*; thus, they are not suitable for the specific diagnosis and epidemiological investigation of these species. Consequently, it is necessary to develop a high-performance technique that offers the possibility to simultaneously detect multiple *Babesia* spp.

Compared with other molecular diagnostic methods, qPCR-HRM has several advantages, namely low cost, high accuracy and rapidity; the same advantages as multiple qPCR based on the use of species-specific probes; and no sequencing or gel electrophoresis is required to analyze the PCR products, thus potential laboratory contamination of PCR products is avoided [56]. In some studies, HRM technology has been applied for the detection of pathogens, and it has been observed that HRM targeting the 18S rRNA gene is a highly reliable strategy for detecting and differentiating species. Bakheit et al. distinguished *Theileria equi* and *Babesia caballi* by HRM [57], Chua et al. explored HRM as a technique for the simultaneous detection of five *Plasmodium* species infecting humans [58] and recently Keatley et al. performed species-level identification of *Trypanosome* infections in Australian wildlife [59]. Rojas et al. also used HRM-qPCR to detect and quantify *Spirocercalupi* in fecal samples from dogs with spirocercosis [60]. Additionally, Wang et al. used HRM to differentiate four *Babesia* species causing bovine babesiosis [47]. The mPCR–HRM assay developed by Bielicka et al. also offers the possibility to detect *B. microti*, *B. divergens*, *B. venatorum* and *B. canis* but requires three pairs of primers for multiple reactions [61], which is more time-consuming than the method proposed herein. The time required to run a sample using the qPCR-HRM method we report here, including amplification curve detection and melting curve analysis, was 1 h and 10 min. The previously reported discrimination criteria for HRM are *T*_*m*_ and melting curve. In addition to these two criteria, we used the GCP value for auxiliary identification in this study.

Since the difference in *T*_*m*_ is directly related to differences in the sequence of the entire target region, once the *T*_*m*_ value and the shape of the melting curve change, misidentification of the target species will result. In this study, HRM primers provided sufficient *T*_*m*_ differences among the five Babesia species. For reliable discrimination, *T*_*m*_ values > 0.25° C are considered to be indicative of different species [47]. The amplicons of *B. duncani* and *B. motasi* *hebeiensis* have the lowest and highest *T*_*m*_, respectively, with a difference of about 1.7 °C. There was no overlap between the *T*_*m*_ range and melting curve characteristics of the five *Babesia* species, which could be accurately distinguished by HRM analysis. Considering the practicability of the qPCR-HRM in the diagnosis of babesiosis, the designed primers ensure high interspecific specificity of the method. Additionally, owing to the high homology existing between the different isolates, the *T*_*m*_ obtained from the prepared plasmid standard showed applicability for distinguishing all the known sequences of targeted species. These results lead to the conclusion that this method is highly versatile for different isolates of the same species worldwide. During the melting curve analysis, the GCP of the automatically invoked results was 90–100%, and the GCP provided strong support for the results of species discrimination.

Wilson et al. used a droplet digital PCR to discriminate between *B. microti* and *B. duncani*, a method which can detect 10 gene copies in a reaction volume [35]. However, the detection limit of our newly developed qPCR-HRM method was one copy for *B. microti* and 10 copies for *B. duncani*. Stanley et al. used cobas *Babesia* (Roche Molecular Systems, Inc.) to detect *B. duncani*, *B. microti*, *B. divergens* and *B. venatorum*, and the detection limits for these four *Babesia* species were reported to range from 6.1 to 50.2 iRBCs/ml [36]. Nevertheless, because the number of DNA copies is the target unit of qPCR-HRM detection, the results of the present study cannot be compared with the sensitivity of the determination developed by Stanley et al. using iRBCs/ml as the measurement unit.

The clinical detection performance of the qPCR-HRM assay was evaluated using clinical specimens and laboratory-infected animal specimens. The results strongly suggest that the assay has the same ability to accurately diagnose *Babesia* as currently available techniques. *Babesia divergens* infection was detected in clinical samples obtained from patients with a history of tick bites. This is consistent with the results previously reported by Wang et al. based on the use of nested PCR [48]. The detection results of blood samples from hamsters infected with *B. duncani* showed that the sensitivity of the qPCR-HRM method to detect *B. duncani* was significantly higher than that of microscopy, and the detection performance was comparable to that of nested-qPCR. Using the qPCR-HRM method, not all of the targeted sequences of samples can be successfully amplified, which is affected by the sensitivity of the diagnostic method. Considering that DNA samples were only extracted from 200 μl of blood, which can lead to false negative results when parasitemia in these samples is extremely low or DNA extraction is improperly performed. Therefore, even if the qPCR-HRM diagnostic assay is relatively sensitive, DNA extraction is a key factor in determining the diagnostic accuracy.

The benefits of the method proposed here notwithstanding, it has some limitations. For example, it does not allow for quantitative detection. Another limitation of this study is that fewer specimens of *Babesia* human cases were collected, and there was a lack of performance data on the applicability of the designed qPCR-HRM assay in clinical practice. In the future, it will be necessary to optimize this technology, such as, for example, by adding species-specific molecular probes, to ensure a higher specificity and detection using more positive clinical specimens.

## Conclusions

The qPCR-HRM assay developed in this study is a promising tool for the simultaneous identification of five human-infecting *Babesia* spp. using only one primer pair. This strategy constitutes an improvement on traditional methods, which are characterized by a low detection performance and can only detect individual species. This assay may be a practical and potential alternative for the rapid and accurate diagnosis of *Babesia* infection, as it also offers the possibility to detect species with low prevalence, such as *B. crassa–*like and *B. motasi* *hebeiensis*. Furthermore, the proposed qPCR-HRM assay can also be applied for blood screening, especially in areas where babesiosis is endemic.

### Supplementary Information

**Additional file 1: Fig. S1.** Sequence alignment and homology analysis of five *Babesia* isolates published in different regions. The two ends of the common sequence are the binding sites of the forward and reverse primers, respectively. **a,** *B. duncani*; **b,** *B. microti*; **c,** *B. divergens*; **d,** *B. crassa-*like; **e,** *B. motasi* *hebeiensis*.

**Additional file 2: Fig. S2** Information on isolates of *Babesia* from different regions used for sequence alignment. **a,** *B. duncani*; **b,** *B. microti*; **c**, *B. divergens*; **d,** *B. crassa-*like; **e**, *B. motasi* *hebeiensis*.

**Additional file 3: Fig. S3** Evaluation of primer efficiency at various annealing temperatures. **a,** 54 °C; **b,** 55 °C; **c,** 56 °C. Lanes: * M,* marker;* 1,* *B. duncani*;* 2,* *B. microti*;* 3,* *B. divergens*;* 4,* *B. crassa-*like;* 5*, *B. motasi* *hebeiensis*;* C,* negative control.

## Data Availability

All the data generated or analyzed in this study are included in the published article. All authors ae data for publication.

## Abbreviations

HRM: High-resolution melting
GCP: Genotype confidence percentage
LVRI: Lanzhou Veterinary Research Institute
ME: Microscopic examination
qPCR: Real-time PCR
*T*_m_: Melting temperature
TTB: Transfusion-transmitted babesiosis
VVBD: Vectors and Vector-Borne Diseases Laboratory
