# Supplementary figures and images for: A high-resolution melting approach for the simultaneous differentiation of five human babesiosis–causing Babesia species
Source: Parasit Vectors. 2023 Aug 28;16:299. doi: 10.1186/s13071-023-05839-5 (PMC10463647; doi:10.1186/s13071-023-05839-5)

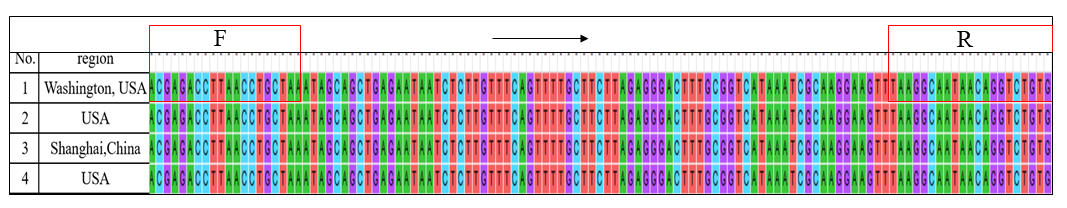
(a) *B. duncani*


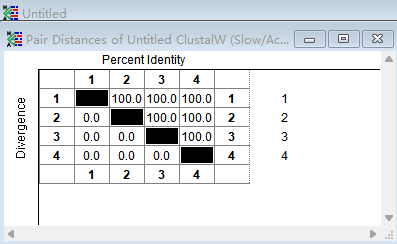


(b) *B. microti*

*
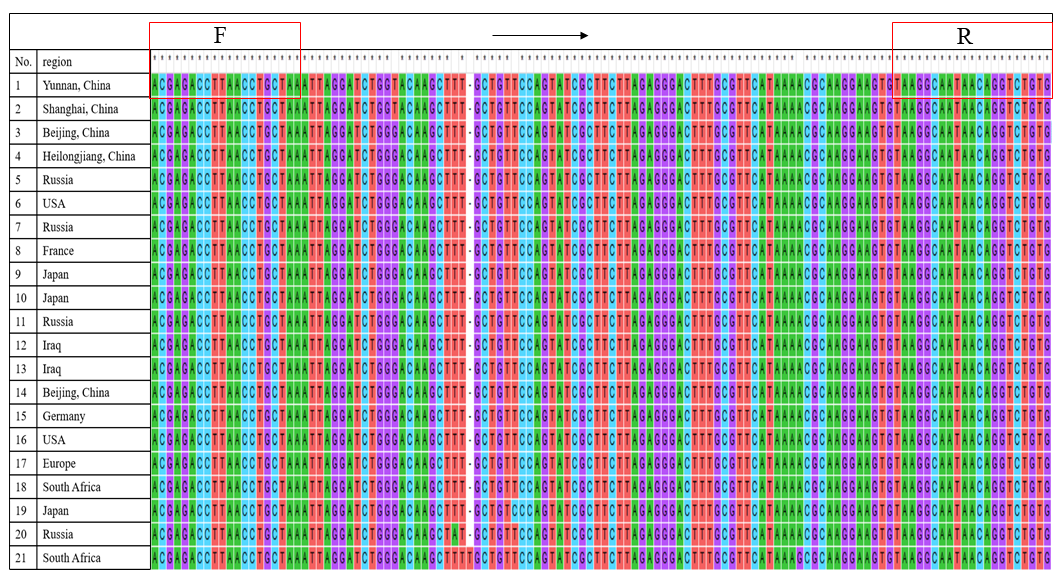
*

*
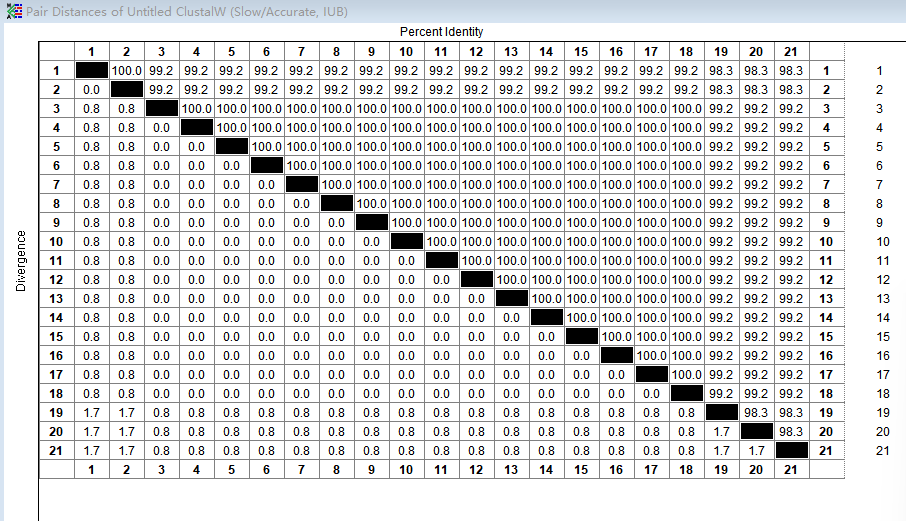
*

(c) *B. divergens*

*
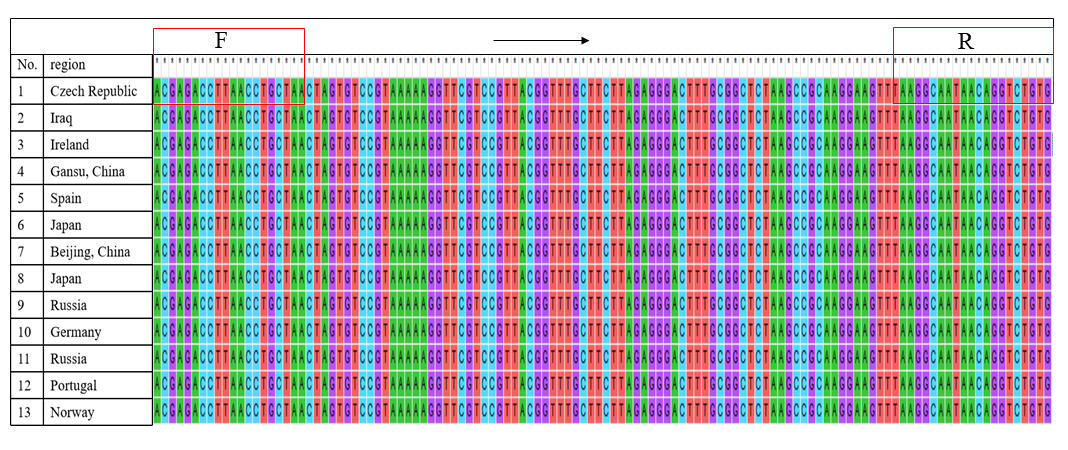

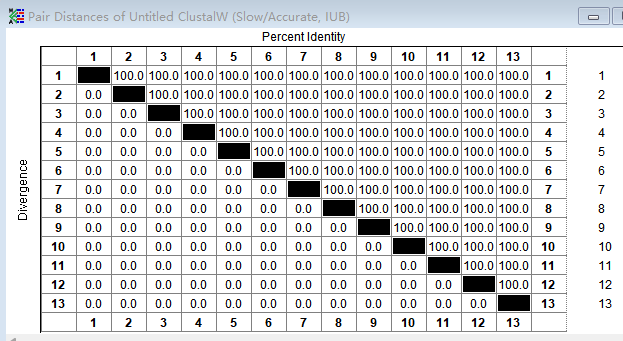
*

(d) *B. crassa-*like


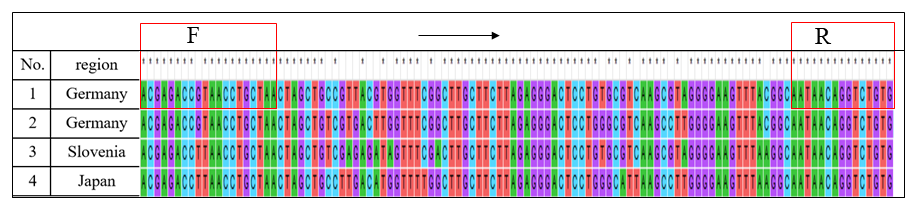


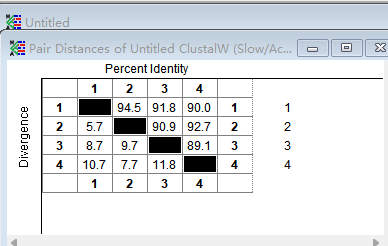


(e) *B. motasi* *hebeiensis*


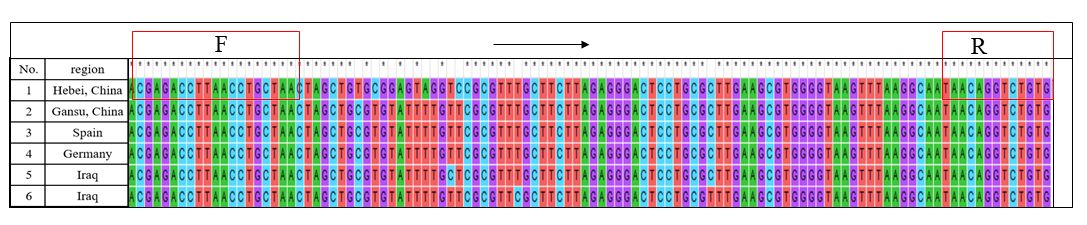


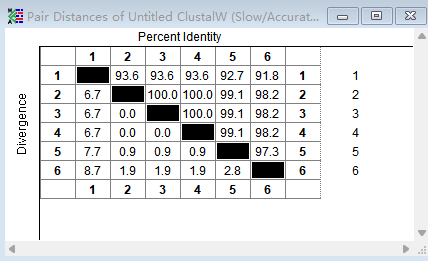

Supplement: Supplementary file 1 — Additional file 1: Fig. S1. Sequence alignment and homology analysis of five Babesia isolates published in different regions. The two ends of the common sequence are the binding sites of the forward and reverse primers, respectively. a, B. duncani; b, B. microti; c, B. divergens; d, B. crassa-like; e, B. motasi hebeiensis. [file 13071_2023_5839_MOESM1_ESM.docx]

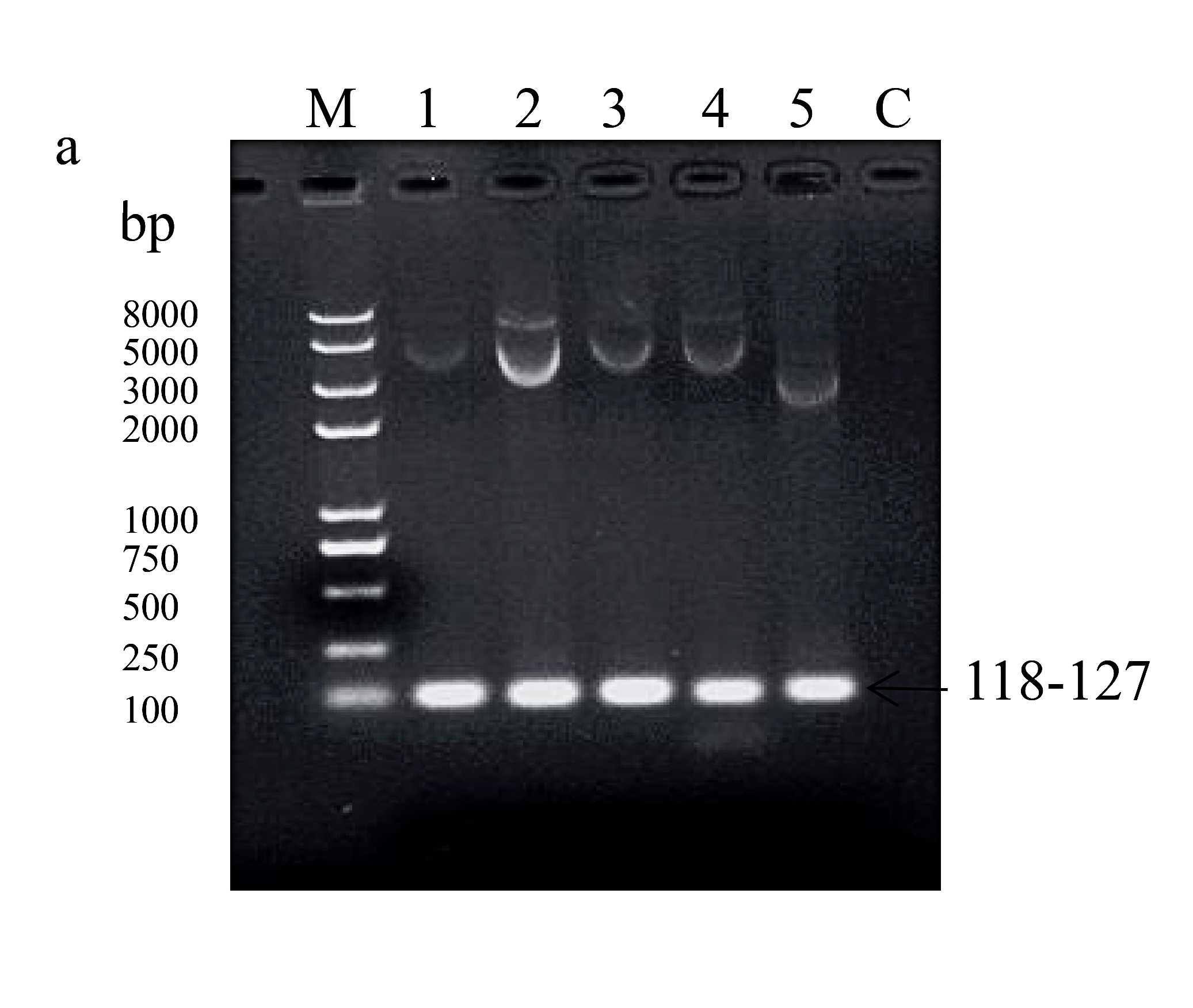


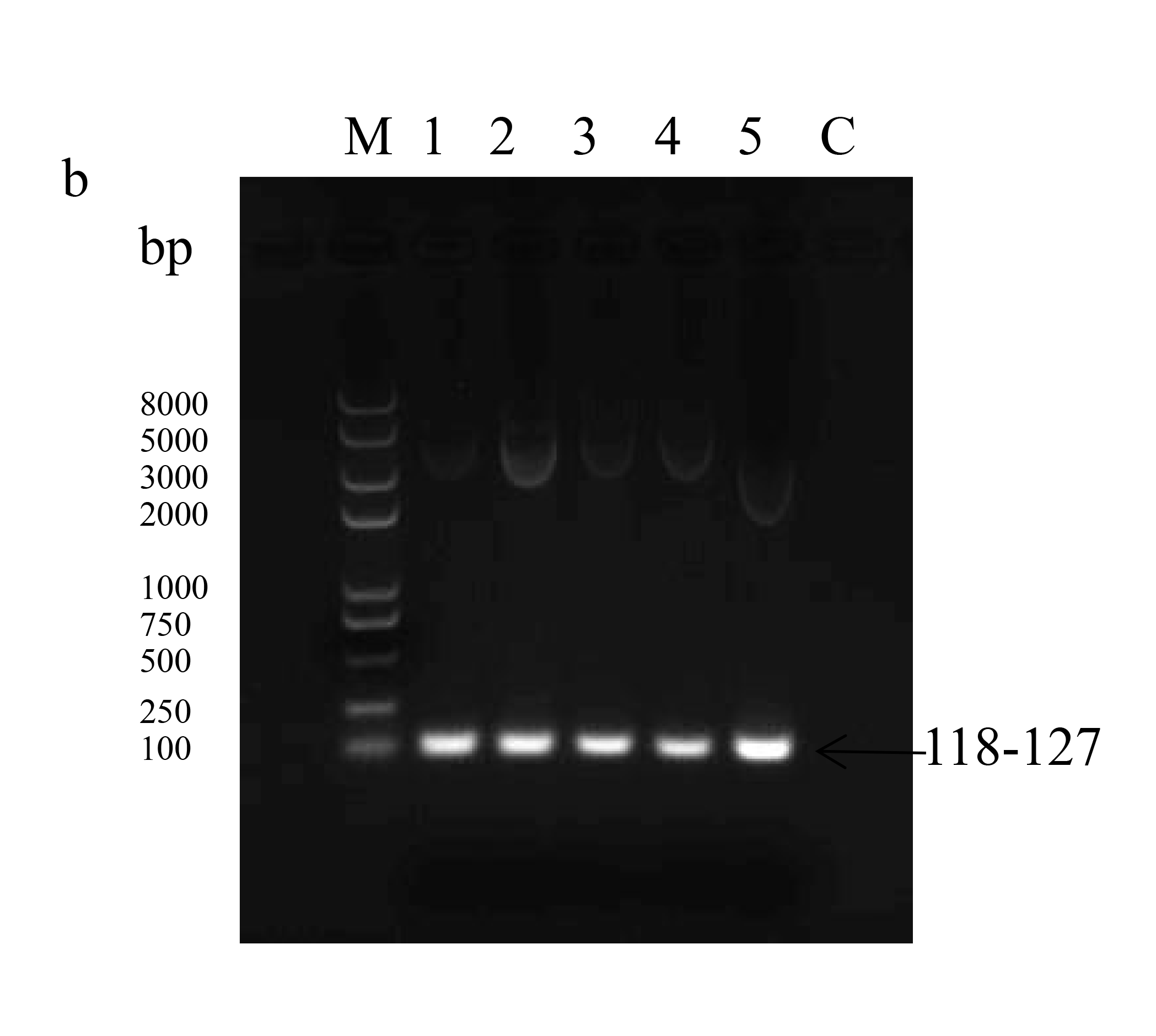


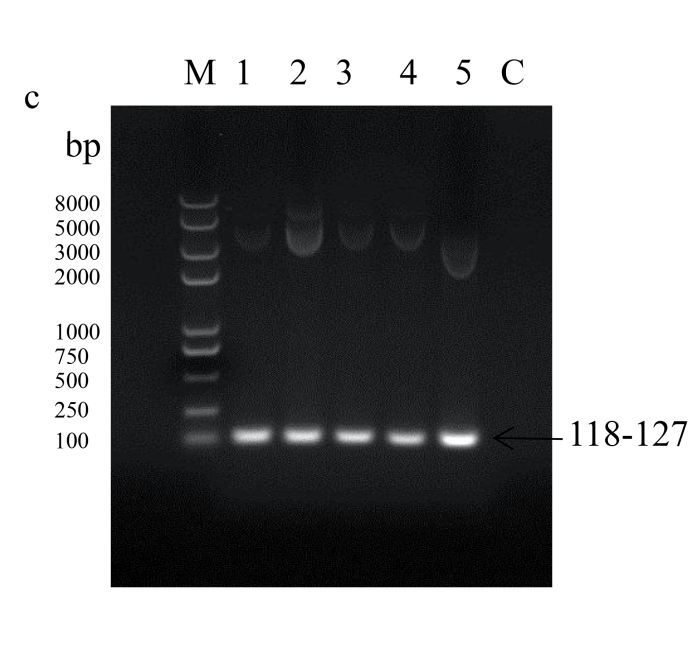

Supplement: Supplementary file 3 — Additional file 3: Fig. S3 Evaluation of primer efficiency at various annealing temperatures. a, 54 °C; b, 55 °C; c, 56 °C. Lanes: M, marker; 1, B. duncani; 2, B. microti; 3, B. divergens; 4, B. crassa-like; 5, B. motasi hebeiensis; C, negative control. [file 13071_2023_5839_MOESM3_ESM.docx]
