# Supplementary material for: A high-resolution melting approach for the simultaneous differentiation of five human babesiosis–causing Babesia species
Source: Parasit Vectors. 2023 Aug 28;16:299. doi: 10.1186/s13071-023-05839-5 (PMC10463647; doi:10.1186/s13071-023-05839-5)
Supplement: Supplementary file 2 — Additional file 2: Fig. S2 Information on isolates of Babesia from different regions used for sequence alignment. a, B. duncani; b, B. microti; c, B. divergens; d, B. crassa-like; e, B. motasi hebeiensis. [file 13071_2023_5839_MOESM2_ESM.docx]

(a) *B. duncani*

| Number | Name of isolates | GenBank acc.numbers | Country |
| --- | --- | --- | --- |
| 1 | *Babesia duncani* isolate BAB2 | HQ285838 | Washington, USA |
| 2 | *Babesia duncani* isolate WA1 | MH333111 | USA |
| 3 | *Babesia duncani* isolate Bdu | KX008042 | Shanghai, China |
| 4 | *Babesia duncani* isolate BAB1615 | HQ289870 | USA |

(b) *B. microti*

| Number | Name of isolates | GenBank acc.numbers | Country |
| --- | --- | --- | --- |
| 1 | *Babesia microti* clone YD86 | KY649343 | Yunnan,China |
| 2 | *Babesia microti* isolate TC-2012-B1 | KF410824 | Shanghai, China |
| 3 | *Babesia microti* strain HLJ552 | KU204797 | Beijing, China |
| 4 | *Babesia microti* strain HLJ44 | KU204793 | Heilongjiang, China |
| 5 | *Babesia microti* isolate RUS/Alt14-1578-Ipv | KX987863 | Russia |
| 6 | *Babesia microti* | LC127372 | USA |
| 7 | *Babesia microti* clone Omsk-vole190_2015 | KU955529 | Russia |
| 8 | *Babesia microti* strain RI chromosome III | LN871598 | France |
| 9 | *Babesia microti* | LC005760 | Japan |
| 10 | *Babesia microti* | LC005752 | Japan |
| 11 | *Babesia microti* isolate Irk-Ip332 | KJ486556 | Russia |
| 12 | *Babesia microti* clone SuAt-Baghdad-11 | MZ442350 | Iraq |
| 13 | *Babesia microti* clone SuAt-Baghdad-8 | MZ442347 | Iraq |
| 14 | *Babesia microti* isolate 14-DC-9 | MT423327 | Beijing, China |
| 15 | *Babesia microti* isolate Jena/Germany | EF413181 | Germany |
| 16 | *Babesia microti* isolate Gray | AY693840 | USA |
| 17 | *Babesia microti* | AB085191 | Europe |
| 18 | *Babesia microti* | U09833 | South Africa |
| 19 | *Babesia microti* | LC005753 | Japan |
| 20 | *Babesia microti* isolate Ubl-104 | AY943958 | Russia |
| 21 | *Babesia microti* clone 46Cat02_10b | MK095343 | South Africa |

(c) *B. divergens*

| Number | Name of isolates | GenBank acc.numbers | Country |
| --- | --- | --- | --- |
| 1 | *Babesia divergens* isolate D86 | MG344781 | Czech Republic |
| 2 | *Babesia divergens* isolate IQKDI-Cattle No.3 | MN124166 | Iraq |
| 3 | *Babesia divergens* TK | LC477143 | Ireland |
| 4 | *Babesia divergens* clone x36-1 | MK256977 | Gansu,China |
| 5 | *Babesia divergens* isolate Spanish_2 | MG944238 | Spain |
| 6 | *Babesia divergens* | LC279018 | Japan |
| 7 | *Babesia divergens* strain HLJ216 | KU377437 | Beijing,China |
| 8 | *Babesia divergens* | AB975389 | Japan |
| 9 | *Babesia* cf*. divergens* clone Omsk-Ip643 | KU955533 | Russia |
| 10 | *Babesia divergens* isolate R105 | KM657258 | Germany |
| 11 | *Babesia* cf. *divergens* isolate Kh-Ip68 | KJ486559. | Russia |
| 12 | *Babesia divergens* isolate Tapada_5 | OL442191 | The Portuguese Republic |
| 13 | *Babesia* cf. *divergens* isolate DP-1578 | JX042329 | Norway |

(d) *B. crassa-*like

| Number | Name of isolates | GenBank acc.numbers | Country |
| --- | --- | --- | --- |
| 1 | *Babesia* cf. *crassa* GU184 | AY260177 | Germany |
| 2 | *Babesia crassa* | AY260176 | Germany |
| 3 | *Babesia crassa* | MK240324 | Slovenia |
| 4 | *Babesia* cf. *crassa* | LC536169 | Japan |

(e) *B. motasi* *hebeiensis*

| Number | Name of isolates | GenBank acc.numbers | Country |
| --- | --- | --- | --- |
| 1 | *Babesia sp.* Hebei-2005 | DQ159074 | Heibei, China |
| 2 | *Babesia motasi* isolate SH. B5 F2 | MN548430 | Gansu, China |
| 3 | *Babesia motasi* | AY533147 | Spain |
| 4 | *Babesia motasi* | AY260179 | Germany |
| 5 | *Babesia motasi* isolate SH. B1F2 | MN551068 | Iraq |
| 6 | *Babesia motasi* isolate SH. B3 F2 | MN548425 | Iraq |
